# A Modified Tridecapeptide Probe for Imaging Cell Junction

**DOI:** 10.3390/molecules29051003

**Published:** 2024-02-25

**Authors:** Jingrui Li, Yuhan Wu, Chunyu Liu, Shu Zhang, Xin Su, Songbo Xie, Fengtang Yang

**Affiliations:** 1 School of Life Sciences and Medicine, Shandong University of Technology, Zibo 255000, China; lcy2804917356@163.com (C.L.); zs19862576280@163.com (S.Z.); m19553336020@163.com (X.S.); xiesongbo@sdnu.edu.cn (S.X.); 2 Center for Cell Structure and Function, Shandong Provincial Key Laboratory of Animal Resistance Biology, Collaborative Innovation Center of Cell Biology in Universities of Shandong, College of Life Sciences, Shandong Normal University, Jinan 250014, China; 15154105683@163.com

**Keywords:** cell junction, Cx43-derived peptide, cell-penetrating peptide, ZO-1

## Abstract

Cell junctions, which are typically associated with dynamic cytoskeletons, are essential for a wide range of cellular activities, including cell migration, cell communication, barrier function and signal transduction. Observing cell junctions in real-time can help us understand the mechanisms by which they regulate these cellular activities. This study examined the binding capacity of a modified tridecapeptide from Connexin 43 (Cx43) to the cell junction protein zonula occludens-1 (ZO-1). The goal was to create a fluorescent peptide that can label cell junctions. A cell-penetrating peptide was linked to the modified tridecapeptide. The heterotrimeric peptide molecule was then synthesized. The binding of the modified tridecapeptide was tested using pulldown and immunoprecipitation assays. The ability of the peptide to label cell junctions was assessed by adding it to fixed or live Caco-2 cells. The testing assays revealed that the Cx43-derived peptide can bind to ZO-1. Additionally, the peptide was able to label cell junctions of fixed cells, although no obvious cell junction labeling was observed clearly in live cells, probably due to the inadequate affinity. These findings suggest that labeling cell junctions using a peptide-based strategy is feasible. Further efforts to improve its affinity are warranted in the future.

## 1. Introduction

Cell junctions are specialized structures located at specific domains of the cell membrane, consisting of gap junctions (GJs), tight junctions, adherence junctions and desmosomes, allowing cells to survive and proliferate. These junctions control key processes in multicellular development, including cell-to-cell communication, cell communication with the extracellular environment, tissue integrity and homeostasis and acting as a site for protein–protein interactions to regulate signaling pathways [1]. In order to carry out these functions, cell junctions dynamically reorganize their structure to respond to the needs of cells. Although the main mechanism regulating the architecture and functioning of cell junctions has been thoroughly examined through cell junction imaging of immunofluorescence staining [2,3] or electrochemiluminescence (ECL) microscopy [4] or fluorescent protein (FP), these technological tools are inherently limited in their performance. Careful determination and testing of the quality and concentration of both primary and secondary antibodies are necessary to achieve a high signal-to-background ratio in immunofluorescence staining [5]. Another limitation is the high concentration of the antibody used during incubation, which can prevent accurate localization of the immune complexes. The materials and step-by-step methods are expensive and time consuming [6]. A major limitation in the development of ECL microscopy is the dramatic decrease in the ECL signal observed when recording successive ECL images [7]. The FP cannot exclude the fact that the large size of an FP moiety can interfere with the dynamic assembly properties of cell junctions. Furthermore, chimeric proteins cannot be separated from the cumbersome means of genetic engineering. Unlike the limitations of fluorescent proteins, peptides offer the advantages of having smaller sizes and being easily synthesized. Hence, the establishment of a simplistic, petite molecule is necessary for tracking cell junction behaviors because it has the advantages of having a smaller size, being easily synthesized, offering low-cost experimental manipulation, being easy and quick to use, and providing the possibility of observing cell junctions in real time, which is imperative to understand the intricacies of cell junctions and their regulatory mechanism.

Tight junctions are the most apical of the cell junctions. They consist of transmembrane proteins called claudins and occludins interacting with cytoplasmic proteins like the ZO-1 protein. Indeed, the ZO-1 antibody can be used for the immunostaining of cell–cell junctions [8], but immunofluorescence staining has the limitations described above. The ZO-1 protein contains three PDZ domains, which are modular protein-binding motifs found in various organisms and play a crucial role in scaffolding protein complexes. The second PDZ domain (PDZ2) binds to the C-terminal cytoplasmic domain of connexins, and the crystal structure of the PDZ2 domain reveals the reconstitution of a peptide-binding groove [9].Therefore, we choose connexins to analyze the ZO-1-binding peptide for the recognition unit of labeling cell junctions. Cx43 is a connexin protein and widely expressed in various tissues and organs, including the heart, brain, and skin. The C-terminal cytoplasmic domain of Cx43 has been shown to interact with the PDZ2 domain of ZO-1. The specific residues involved in the interaction between Cx43 and PDZ2 vary among different connexins, but they generally include a conserved ASSR/K sequence. Based on the structural basis of the interaction between ZO-1 and Cx43, we aimed to create a fluorescent peptide derived from Cx43 and delivered it into cells through a strategy involving a cell-penetrating peptide.

## 2. Results

### 2.1. Design of a Cx43-Derived Tridecapeptide for Monitoring Cell Junction

**Figure 1:**
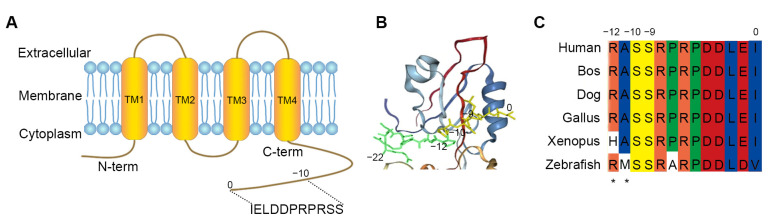
Cx43-derived tridecapeptide. (**A**) Domain structure of Cx43. The Cx43 protein includes four transmembrane domains (TM), an intracellular amino acid domain and intracellular amino acid domain. The recognition unit is from C-term. (**B**) The conformational image represents the docking analysis between the last twenty-three residues (−22~0) from carboxyl tail region (yellow) of Cx43 and the PDZ domain protein of ZO-1. The final three residues of the Cx43 peptide attach to the groove, and Ser (−9) and Ser (−10) closely interact with the pocket. (**C**) Amino acid sequence alignment of the C-terminal tail (−12~0) of Cx43 from different species. Ser (−9) and Ser (−10) of Cx43 are highly conserved and regulate the Cx43/ZO-1 complex. To guarantee binding, the Cx43 peptide was elongated by two residues. This peptide is known as the recognition unit of ZO-1 for labeling cell junction. The asterisks indicate the two expanded amino acids.

PDZ domains are protein-interacting modules that attach to short peptide fragments of target proteins in eukaryotic proteomes [10,11,12]. Zonula occludens-1 (ZO-1) is a tight junction protein that plays a crucial role in the formation and maintenance of tight junctions [13,14]. Jia Chen et al. [9] discussed the domain-swapped dimerization of ZO-1 PDZ2 and its role in generating specific and regulatory connexin43 (Cx43) binding sites. Based on the finding, we firstly analyzed the carboxyl tail region of Cx43 (Figure 1A) and the PDZ2 domain of ZO-1 by docking using a molecular docking procedure GalaxyWEB (https://galaxy.seoklab.org/index.html. accessed on 12 October 2023). The result showed that the PDZ domain-swapped assembly creates a symmetric pocket binding to the canonical carboxyl tail (−22~0, the last twenty-three residues) of Cx43 (Figure 1B). Moreover, the Cx43/ZO-1 complex can be further regulated by the phosphorylations of Ser (−9) and Ser (−10) in Cx43 [9], as Ser (−9) and Ser (−10) are substrates of various kinases, such as PKC and Akt [15,16,17]. Here, we extended the Cx43 peptide by the asterisked two residues (−12~0, the last thirteen residues). The tridecapeptide fragments were highly conserved in different species (Figure 1C) and further confirmed that the peptide can be used as a recognition unit of ZO-1 for labeling cell junctions, just as the 17 conserved amino acids act as a recognition unit of F-actin for labeling microfilaments [18,19].

### 2.2. Modified Tridecapeptide Can Bind ZO-1 and Label Cell Junction of Fixed Cells

**Figure 2:**
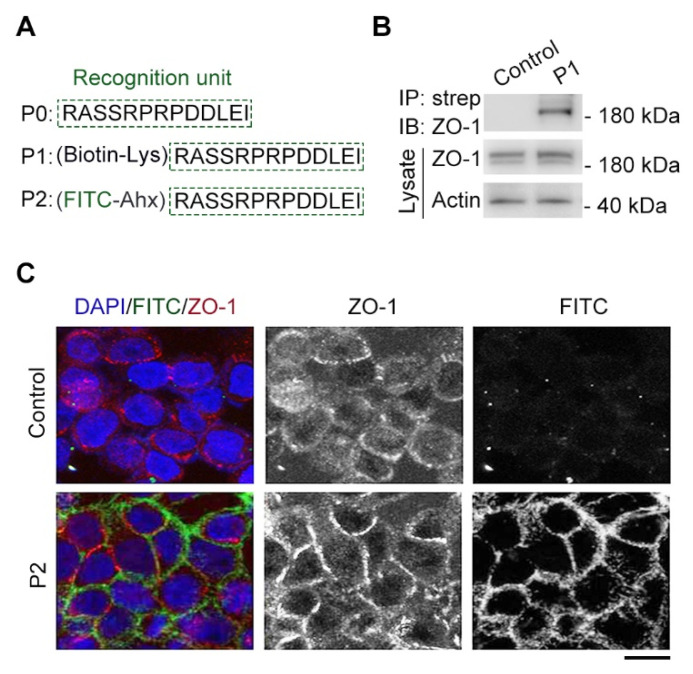
Cx43-derived tridecapeptide can bind ZO-1 and label cell junction. (**A**) The components of synthesized Cx43-derived peptide, which contains a recognition unit (P0), an introduced lysine (Lys) conjugated to biotin (P1), an introduced aminohexanoic (Ahx) acid-conjugated fluorescein isothiocyanate (FITC) dye (P2). (**B**) Gel images of streptavidin–biotin pulldown and immunoblotting reveal the binding between P1 and tight junction protein ZO-1. P1: (biotin–Lys) RASSRPRPDDLEI, strep: streptavidin. For the control group, a biotin-Lys (without the recognition unit) was used. (**C**) The fixed cells were subjected to immunofluorescence staining by ZO-1 primary antibody and Alexa Fluor 568-conjugated secondary antibody. (red) Then, the fixed cells were subjected to fluorescent probe labeling by P2 (green) incubation for half an hour and away from light. Nuclei were stained with DAPI (blue). P2: (FITC-Ahx) RASSRPRPDDLEI, DAPI: 4′,6-diamidino-2-phenylindole. For the control group, an FITC-Ahx (without the recognition unit) was used. Scale bars, 5 μm.

The tridecapeptide RASSRPRPDDLEI motif was referred to P0 as the recognition unit of cell junctions and modified with biotin through introducing lysine (Lys) to test the binding between the tridecapeptide motif with the ZO-1 protein by using the streptavidin pulldown technique [20] (Figure 2A). We performed a transfection of HEK293 cells using the constructed ZO-1 plasmid for ZO-1 protein overexpression, and after 24 h, we incubated the synthesized biotin-modified peptide (P1) in the cell lysates at room temperature for 4 h. Then, the binding capacity of P1 with the ZO-1 protein was confirmed through streptavidin–biotin pulldown and immunoblotting assays. As expected, biotinylated peptide could pull ZO-1 protein from cell lysates, whereas binding was not detected in the control group with only biotin (Figure 2B), suggesting the tridecapeptide RASSRPRPDDLEI motif could be used as a recognition unit of ZO-1. 

Next, the tridecapeptide was modified with fluorescein isothiocyanate (FITC) for the fluorescent labeling of cell junctions and an aminohexanoic (Ahx) acid was introduced as a protective group [19,21] to improve the stability of FITC (Figure 2A). We then examined the synthesized an FITC-modified peptide (P2) instead of using an antibody to label cell junctions via an immunofluorescence assay [3,8]. There was some degree of colocalization between the FITC-modified peptide labeling and the tight junction component, ZO-1, antibody staining, whereas colocalization was not detected in the control group with only FITC-Ahx (Figure 2C), suggesting the FITC-modified tridecapeptide could label cell junctions of fixed cells.

### 2.3. The Tridecapeptide Can Localize at Cell Junction of Live Cells by Plasmid Expressing Rather Than Using the Membrane-Penetrating Peptide

**Figure 3:**
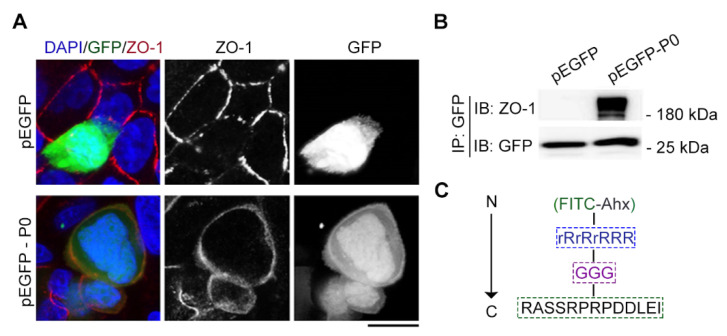
Cx43-derived tridecapeptide can localize at cell junction of live cells by binding ZO-1. (**A**) The images represent immunofluorescence staining (red) tight junction for ZO-1 antibody, location of the tridecapeptide (P0) fused with GFP (green), and staining nuclei (blue) for DAPI. P0: RASSRPRPDDLEI, DAPI: 4′,6-diamidino-2-phenylindole. For the pEGFP group, an empty plasmid vector (without the P0) was used. Scale bars, 5 μm. (**B**) Gel images of immunoprecipitation and immunoblotting reveal the binding between P0 and tight junction protein ZO-1. P0: RASSRPRPDDLEI. For the pEGFP group, an empty plasmid vector (without the P0) was used. (**C**) The component of fluorescent Cx43-derived peptide which contains a fluorescein isothiocyanate (FITC) dye at the N-terminus, a cell-penetrating peptide (rR)_3_R_2_, a spacer (GGG), and a ZO-1 recognition unit (RASSRPRPDDLEI) at the C-terminus. Ahx, e-aminocaproic acid; r denotes D-arginine; and R denotes L-arginine.

Next, we examined whether the tridecapeptide could localize at the cell junction of live cells. We successfully constructed an expression plasmid incorporating GFP for the tridecapeptide and then transfected it into Caco-2 cells with Lipofectamine 3000 (Thermo Fisher Scientific, Waltham, MA, USA). Strikingly, a microscopic observation of the live cell [22] showed that the tridecapeptide was also able to localize at the cell junction of live cells, which can be confirmed by the co-localization of the ZO-1 antibody staining after fixation of the cells (Figure 3A). To further validate the binding between the tridecapeptide and the cell junction protein ZO-1, we conducted an immunoprecipitation assay based on beads coupled with the GFP antibody. Consistent with microscopically observed co-localization results, the tridecapeptide fused with GFP could pull the ZO-1 protein from cell lysates, whereas binding was not detected in the control group with only GFP (Figure 3B).

**Figure 4:**
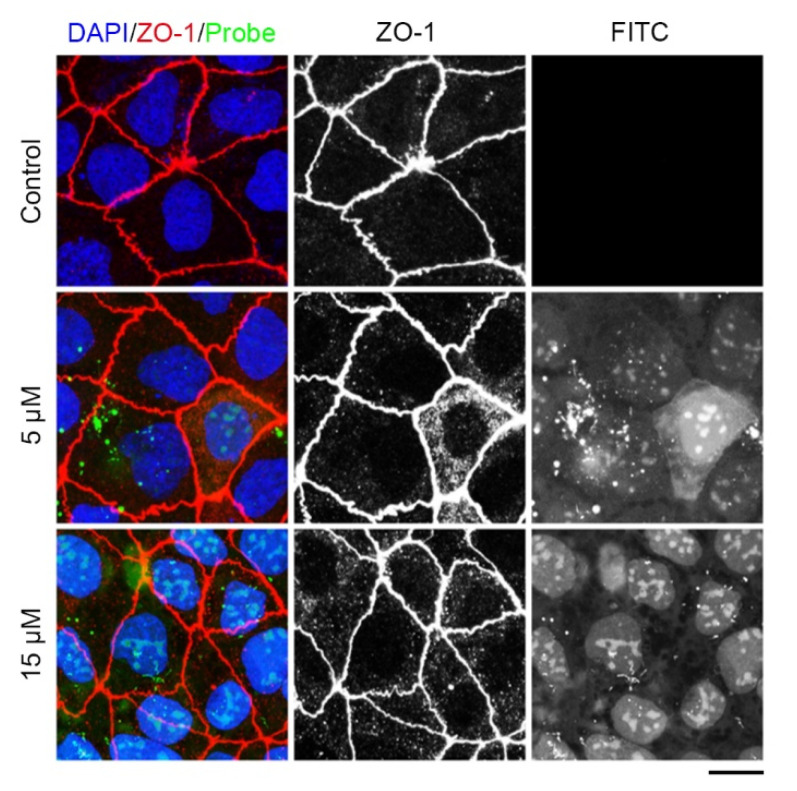
Examination of the tridecapeptide labeling cell junction in live cells. Caco-2 cells were treated with 5 μM, 15 μM of the tridecapeptide, or control peptide, together with Hoechst 33342, and 30 min later, the cells were imaged on a Leica SP8 confocal microscope. The images represent immunofluorescence staining (red) tight junction for ZO-1 antibody, location (green) of the tridecapeptide in live cells, and staining nuclei (blue) for DAPI. DAPI: 4′,6-diamidino-2-phenylindole. For the control group, an FITC fluorescence-modified peptide (without the cell-penetrating peptide) was used. Scale bars, 5 μm.

We supplemented an oligoarginine (rR)_3_R_2_ (r: D-Arg, R: L-Arg), a well-established cell-penetrating peptide [23,24], in order to more quickly transport the tridecapeptide into cells. To maintain the natural conformation of the tridecapeptide, three glycine residues were introduced as a spacer and to improve the stability of FITC. Ahx acid was introduced as a protective group (Figure 3C). Then, we synthesized the designed fluorescent polypeptide and the control peptide that did not contain the cell-penetrating peptide. Last, we investigated whether the fluorescent polypeptide could effectively enter into live cells and label cell junctions. The cell-penetrating peptide (rR)_3_R_2_ was able to successfully deliver the fluorescent polypeptides into Caco-2 cells, whereas the control peptide lacking the cell-penetrating peptide was unable to enter the cells (Figure 4). However, the fluorescent polypeptides were localized to the nucleus instead of the cell junction. After fixing the live cells, immunofluorescence staining with the ZO-1 antibody indicated that cell junctions had been formed, which suggests that the cell junctions had been not yet formed. Given these facts, we speculate that this is probably due to the stronger mutual attraction between the positively charged membrane-penetrating peptide and the nucleus than between the tridecapeptide and the ZO-1 protein. Therefore, in order to label cell junctions of live cells, it is necessary to retrofit the amino acid residues for increasing the tridecapeptide’s affinity to the ZO-1 protein.

## 3. Discussion

Electron microscopy and immunofluorescence staining are currently major methods of observing cell junctions. A major limitation of the electron microscopy is the autofluorescence, which significantly increases background signals and makes the fluorescent imaging of stretched cells difficult [25,26]. Careful determination and testing of the quality and concentration of both primary and secondary antibodies are necessary to achieve a high signal-to-background ratio in immunofluorescence staining. The limitations require the development of an alternative approach. Here, we developed a peptide-based strategy for constructing a cell-permeable fluorescent probe for real-time observations of cell junctions by exploiting the remarkable cytosolic delivery ability of (rR)_3_R_2._ We constructed a peptide-based fluorescent probe, which contained a recognition unit and fluorophores. Our results indicate that the probe can both directly label cell junctions in fixed cells and enter live cells efficiently. The tridecapeptide probe strategy offers several important advantages over immunofluorescence staining or electron microscopy, including having a smaller size, offering low-cost experimental manipulation, being easy and quick to use and providing the possibility of observing cell junctions in real time. Unlike the limitations of fluorescent proteins, peptides offer the advantages of having smaller sizes and being easily synthesized.

The first 17 aa of Abp140 is homologous among close relatives of *Saccharomyces cerevisiae*, which is used as a marker to visualize F-actin structures in eukaryotic cells and tissues [18]. Our previous research showed that a tau-derived homologous motif peptide is able to bind both tubulins and microtubules [24]. In this study, we tested a Cx43-derived homologous tridecapeptide (Figure 1) for its ability to label cell junctions. Our data revealed that the modified tridecapeptide from Cx43 can bind the tight junction protein ZO-1 and label cell junctions of fixed cells (Figure 2). Taken together, the evidence suggests that the homologous motif peptide sequences are critical for the interactions between molecules. The dynamic assembly of cell junctions in live cells is associated with many cellular activities such as cell migration [27]. Alterations in the dynamic assembly of the cell junction impair blood–brain barrier properties, particularly influencing barrier integrity and permeability. Therefore, we tested the Cx43-derived tridecapeptide in live cells and found that the tridecapeptide can localize at the cell junction of live cells by binding ZO-1 (Figure 3), which opens up the possibility of observing the dynamics of cell junctions in real time. Taken together, the modified tridecapeptide from Cx43 binds ZO-1 specifically and labels cell junctions, providing an alternative tool for convenient cell junction labeling without the need of pricey antibodies, which may facilitate studies about cell junctions. Although the modified tridecapeptide-conjugated (rR)_3_R_2_ may not have a satisfactory image in live cells (Figure 4), our data suggest that using a peptide-based strategy for imaging subcellular structures is feasible. Notably, the easy replacement of the recognition unit or cell delivery strategy will greatly expand the possibility of observing cell junctions in live cells. It is recommended that attempts be made to identify novel peptides with higher affinity in the future.

## 4. Materials and Methods

### 4.1. Materials

Antibodies against ZO-1 (33-9100) (Thermo Fisher Scientific, Waltham, MA, USA), β-actin (A5316) (Sigma-Aldrich, St. Louis, MO, USA), and GFP (50430-2-AP) (Proteintech, San Diego, CA, USA) were purchased from the indicated sources. The horseradish peroxidase-conjugated secondary antibodies were purchased from Amersham Biosciences (Amersham, UK). Alexa Fluor 568–conjugated secondary antibody was purchased from Abcam (Cambridge, UK). Nocodazole, 4′,6-diamidino-2-phenylindole (DAPI) and streptavidin beads were purchased from Sigma-Aldrich. Caco-2 cells [American Type Culture Collection (ATCC) number: HTB-37] were cultured in Dulbecco’s modified Eagle’s medium (DMEM) (Thermo Fisher Scientific) supplemented with 10% fetal bovine serum (Biological Industries, BioInd., Migdal HaEmek, Israel).

### 4.2. Peptide Synthesis

Using standard Fmoc chemistry from GenScript (Nanjing, China), Cx43-derived and scrambled control peptides were synthesized via solid-phase methodology. N-terminal fluorescein labelling was carried out according to a previously described method [19]. The synthesized peptides were purified via high-performance liquid chromatography using a C-18 reversed-phase column. They were then analyzed via mass spectrometry, and we tested their solubility using double-distilled water, a phosphate-buffered solution or dimethyl sulfoxide.

### 4.3. Immunoprecipitation and Immunoblotting

Cells were disrupted in the lysis buffer (Cell Signaling Technology) supplemented with a protease inhibitor cocktail (Thermo Scientific). The resulting lysates were sonicated and centrifuged at 15,000 rpm for 20 min at 4 °C. For the immunoprecipitation, the samples were incubated overnight at 4 °C with either streptavidin or agarose beads that were coated in a GFP antibody. The beads were rinsed five times, and the affiliated proteins were analyzed using SDS-PAGE and immunoblotting techniques. For immunoblotting, the proteins were separated using SDS-PAGE and subsequently transferred onto PVDF membranes (Millipore, Burlington, MA, USA). The PVDF membranes were blocked in a solution of 5% fat-free dry milk in tris-buffered saline that contained 0.1% Tween 20. The membranes were blocked prior to incubation with primary and horseradish peroxidase-conjugated secondary antibodies. The proteins of interest were detected using an enhanced chemiluminescent substrate (Thermo Fisher Scientific, Pierce Biotechnology, Waltham, MA, USA).

### 4.4. Fixed Cell Assays

Caco-2 cells were fixed with 4% paraformaldehyde for 30 min. This was followed by permeabilization using 0.5% Triton X-100 (Beyotime, Shanghai, China). The fixed cells then underwent blocking with 4% bovine serine albumin in PBS and incubation with the ZO-1 antibody. Subsequently, the secondary antibodies conjugated with either Alexa Fluor 568 (Thermo Fisher Scientific, Waltham, MA, USA) was applied, and DAPI was used for staining. The FITC-conjugated peptide was added to the fixed cells and incubated for 30 min. After washing three times with PBS, the fixed cells were imaged on a Leica SP8 confocal microscope using an HC PL APO CS2 63x/1.40 oil objective at 1024 × 1024 resolution and 1 × zoom factor. Tile scans were stitched with the Leica LAS AF software (Version 3.0).

### 4.5. Live Cell Assays

Human embryonic kidney (HEK) 293 cells (ATCC number: CRL-1573) and Caco-2 cells (ATCC number: HTB-37) were cultured in the minimum essential medium (provided by Thermo Fisher Scientific) along with 10% fetal bovine serum (provided by Biological Industries) and maintained at 37 °C in a humidified atmosphere with 5% CO_2_ environment. After cell adherence, pEGFP-N1 plasmids expressing the peptide were transfected into cells with the Lipofectamine 3000 (Thermo Fisher Scientific). The culture medium was replaced with fresh DMEM containing 10% FBS after 8 h of transfection. On the other hand, the membrane-penetrating peptide-conjugated peptide was added to the Caco-2 cells and incubated for 30 min. Then, the cells were washed three times with PBS. The nuclei were stained with Hoechst 33342 (Beyotime, Shanghai, China). The cells were imaged on a Leica SP8 confocal microscope using an HC PL APO CS2 63x/1.40 oil objective at 1024 × 1024 resolution and 1 × zoom factor. Tile scans were stitched with the Leica LAS AF software (Version 3.0).

## 5. Conclusions

This study demonstrates that the modified tridecapeptide from Cx43 can bind to the tight junction protein ZO-1 and label cell junctions of fixed cells. Additionally, we found that the Cx43-derived tridecapeptide can localize at the cell junction of live cells by binding ZO-1. The modified tridecapeptide from Cx43 binds ZO-1 specifically and labels cell junctions. This provides a cost-effective alternative to using antibodies for convenient cell junction labeling, which may facilitate studies about cell junctions. Our data suggest that using a peptide-based strategy for imaging subcellular structures is feasible.

## Data Availability

The data presented in this study are available in article and Supplementary Materials.

## Supplementary Materials

The following supporting information can be downloaded at: https://www.mdpi.com/article/10.3390/molecules29051003/s1.
