# Supplementary figures and images for: A Modified Tridecapeptide Probe for Imaging Cell Junction
Source: Molecules. 2024 Feb 25;29(5):1003. doi: 10.3390/molecules29051003 (PMC10935238; doi:10.3390/molecules29051003)

Mass spectral analysis details of fluorescein labelled tridecapeptide.

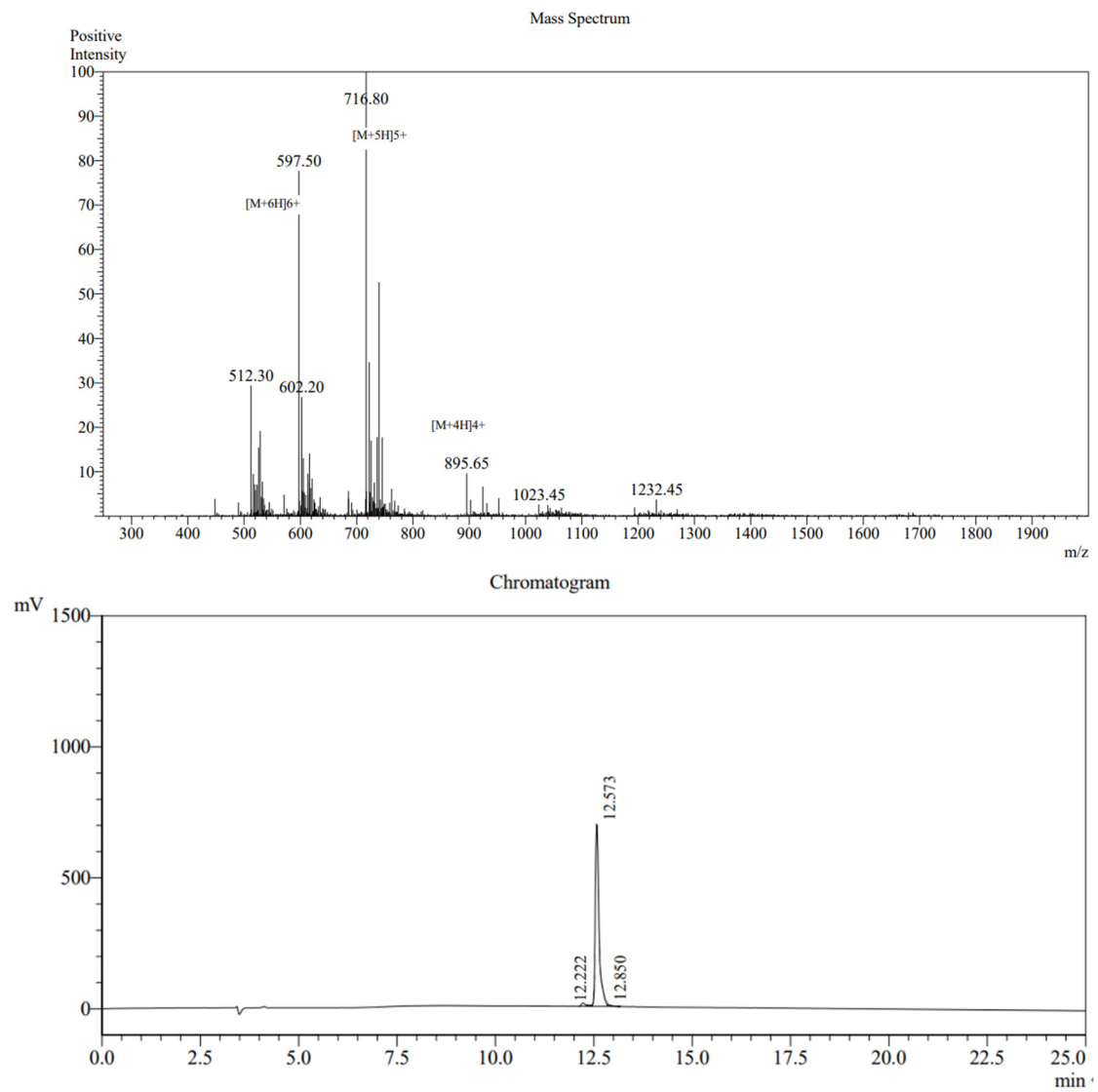

Supplement: Supplementary file 1 [file molecules-29-01003-s001.zip › molecules-2834935-supplementary.pdf]
